# Longer wavelengths in sunlight pass through the human body and have a systemic impact which improves vision

**DOI:** 10.1038/s41598-025-09785-3

**Published:** 2025-07-08

**Authors:** Glen Jeffery, Robert Fosbury, Edward Barrett, Chris Hogg, Marisa Rodriguez Carmona, Michael Barry Powner

**Affiliations:** 1 Institute of Ophthalmology, University College London, 11-43 Bath St, London, EC1V9EL UK; 2 Institute for Environmental Design and Engineering, UCL, London, UK; 3 Centre for Applied Vision Research, City St George’s, University of London, London, UK

**Keywords:** Sunlight, Visual function, Body transparency, Neuroscience, Physiology

## Abstract

Long wavelength red light that can extend beyond the human visual range penetrates deeply through biological tissue. Exposure to these longer wavelengths improves mitochondrial function and ATP production. This can translate to improved physiological performance, particularly in the CNS, including the visual system. Light driven metabolic improvements to regional exposure can impact systemically. Here we show that infrared wavelengths from sunlight can be measured after they pass through the human thorax. We then select a prominent transmitted solar wavelength range (830–860 nm) and deliver this to the thorax of subjects in the lab in controlled 15 min exposures with and without ocular involvement. Clothing reduced wavelength intensity but was not a barrier. These exposures were associated with significantly improved visual function when measured 24 h later even in subjects in which light was blocked from the eyes. Our data show that longer wavelengths of sunlight penetrate through the human body and, consistent with animal studies, have the ability to improve function. While infrared light has been used on targeted tissues, its ability to improve distal tissues in humans has not been explored. This study also highlights the potentially important therapeutic value of sunlight whose longer wavelengths can reach key organs even through clothing and likely promote mitochondrial function counteracting the decline with age and disease.

## Introduction

The sunlight spectrum at ground level ranges from approximately 300 nm – >3000 nm and has been a stable influence on life across evolution, providing the energy to drive evolutionary change. Its composite wavelengths play separate roles in physiological regulation by influencing mitochondria that are key agents in metabolism and ageing. Mitochondria produce ATP, often referred to as the cellular currency of cells in terms of energy production. They have a membrane potential that declines with age and disease resulting in reduced ATP production. This decline is commonly associated with increased reactive oxygen species (ROS) that drives inflammation and can result in cell death and the decline of the organism. This forms the core of the mitochondrial theory of ageing^1^.

Mitochondria are light sensitive. Longer wavelengths (approximately 660–1000 nm) increase mitochondrial membrane potential and ATP production particularly when they have declined with age or disease and can improve performance^2–7^. This is possibly in part due to their absorption by the copper heme in cytochrome c oxidase^8 ^although absorption patterns influencing physiology are likely to be more complex than this alone. Shorter wavelengths (400–450 nm) have the opposite effect, most probably being absorbed by in the Soret band of a porphyrin, resulting in ROS production and reducing mitochondrial function and performance^9^. The differential impact of these spectral regions is highly conserved across species but they do not have hard borders. In sunlight these long and short spectral components have a stable balance present in daylight across evolution.

The outer retina contains more mitochondria than any other tissue, with a high metabolic rate and a concomitant rapid pace of ageing^10^. Consistent with this, in human subjects, long wavelength application results in improved visual function^7^. It also can reduce blood sugars, as increasing mitochondrial function and ATP production requires elevated demand for serum carbohydrates^11^. This is an example of systemic impact because only small regions of the body are exposed to significantly reduce blood sugars. Likewise, improved CNS performance can be obtained when distal regions of the body are exposed^12,13^. This systemic influence may be mediated by cytokine signalling as the complex array of serum cytokines shifts significantly following regional long wavelength light exposure^14^.

Many experiments using longer wavelengths have concentrated on 670 nm which is visible red light. Here we ask if longer wavelengths present in sunlight and that are beyond our visual range that extends approximately between 400 and 700 nm can penetrate deeply through the human body and influence visual function with and without ocular involvement.

## Methods

Experiments were undertaken on 40 Caucasian subjects of both sexes between the ages of 25 to 63 under ethical approval from City St George’s, University of London (ETH2324-0250) and complied with the code of ethics of the world medical association (Declaration of Helsinki). Informed consent was obtained from all subjects. A series of laboratory experiments were undertaken and measurement made of light and its penetrance in the open environment. They were either drawn from the academic community or from those from the commercial lighting, design and architecture community. The experiments measured sunlight and LED light penetrance through the human thorax and hand and its impact on visual function. They also assessed differing light wavelength penetration on clothing.

### Sunlight

Male subjects (*N* = 7. Mean age 37 ± 17) stood with their backs facing clear sunlight in mid-June, with no clothing from the waist up. A metal box 15 cm square and 30 cm long made from 1 mm thick aluminium sheeting was placed against their chest. This was used to house probes from either a spectrometer (Ocean Optics QE65 ) or a radiometer (Interlight ILT5000 Research Radiometer) to measure the wavelength/energy of sunlight passing through the body. With measuring probes placed against the subjects chest the rear opening of the box was packed with aluminium foil to block stray light. Three independent measurements were made of light passing through the thorax with each device over a period of approximately 3 min. These data provided the energy of light passing through the thorax and its spectral profile. Additionally, an infrared sensitive camera was used to image the light and directly visualise its transmission through the thorax. As a control for the identification of potential light absorbers, a separate experiment was undertaken. First, a human hand was imaged over an 850 nm LED source with an infrared sensitive camera to confirm that this wavelength range penetrated the tissue. This was in a male subject aged 75 years. This tissue was approximately 3 cm thick. This wavelength range was used because it was a dominant feature of light passing through the thorax. Second, the spectrum of light through the hand was analysed from a continuum source of a tungsten filament light via an integration sphere. These data were mapped against the known absorbance profiles of water and haemoglobin to determine their contributions to absorbance across the hand.

### LED 850 nm light

Light penetrance was also measured through the thorax from an 850 nm light source in the form of a standard 60 cm X 60 cm ceiling panel containing an array of 3024 850 nm LEDs equally spaced across it giving an energy of 9.18mW/cm^2^ at 50 cm, which was the distance at which subjects were exposed and measurements of transmission made. This was manufactured to our specifications by Light, Power Health UK. 850 nm LEDs had a half power band width of 830 nm and 865 nm in a slightly positively skewed asymmetric distribution. This LED was chosen because its range approximated that of a prominent spectral range of sun light measured passing through the thorax. Measurements of LED light transmission were made in a dark laboratory. In a separate group of subjects, (*N* = 8. Mean age 46 ± 15) the transmission of light through the thorax from the 850 nm panel was measured in a manner similar to the daylight experiments in a darkened room. The 850 nm light could not be seen with the eye unless the subject was close to it and could not be seen at the distance used for exposure in the room. A further group 13 subjects (8 male, 5 female. Mean age 48 ± 20) were also exposed to the panel with the purpose of assessing this exposure on visual function (850 nm group). These data were compared against a further 5 subjects (Male. Mean age 36 ± 9) who had their head wrapped in aluminium foil to differentiate between eye mediated, and body surface mediated effects (850 nm body only group). Additionally, 7 control subjects (Female. Mean age 35 ± 8) were placed in the same position in the room but the 850 nm panels was not turned on, as a sham exposure (Control group). A trial for each subject, consisted of a first visit during which baseline colour contrasts thresholds were measured. Immediately following this, participants were exposed to the 850 nm light or a sham exposure (control) for 15 min at 9 mW/cm^2^. Exposure was to their back with no clothing on their upper body, in a dark room. 24 h later the participants returned, and their colour contrast thresholds were measured a second time.

The assessment of visual function was based on colour contrast detection thresholds. Colour contrast was chosen because with age cone photoreceptors have reduced function mainly due to mitochondrial decline^7^. However, these cells rarely die, making them idea for optical manipulation to improve mitochondrial performance. Each subject had their colour contras measured psychophysically using Chroma test^7^. This is a computer graphics system, where letters of varied contrast are presented binocularly on a high-resolution NEC Spectraview monitor on an isoluminant background at a distance of 1.5 m in a dark room, exactly as employed by Shinhmar et al.^7^. Shinhmar et al. provided the end point X, Y coordinates defining the protan and tritan axis according to the CIE colour space in their Table 2, which were identical to those used here. If subjects correctly identified the presented letter, the contrast in the colour domain was reduced. If subjects incorrectly identified the letter the contrast was increased. This was repeated in multiple tests until detection threshold was determined.

On each trail tritan and protan presentations were interleaved sequentially. Room illumination was approximately 3 lx. Each subject was given a familiarisation trial and then tested on 5 consecutive trials. In each trial approximately 30 letters were presented giving approximately 100–120 test trails per subject. Outcome data are given as thresholds in percentage contrast detection for protan and tritan domains. Because infrared wavelengths can be reflected from surfaces and also penetrate eye lids when closed, 5 subjects had their heads wrapped in aluminium foil to completely block light from the eye during light exposure. 24 h later subjects returned and were retested for colour contrast.

In sunlight and 850 nm LED light subjects were not wearing upper clothing and it is unclear if clothing modifies long wavelength light. Hence, we measured qualitatively the transmission of visible and 850 nm though clothes comprising layered fabrics. This was determined using a camera (a modified Sony α7R with full UV - IR response with appropriate visible and NIR-pass > 760 nm filters) sensitive to both the human visual range and NIR light. The clothing consisted of two sets of three layers: a knitted wool and two layers of polyester/polyamide fabric (a shirt and a T-shirt).

## Results

### Sunlight transmission through the body

**Fig. 1:**
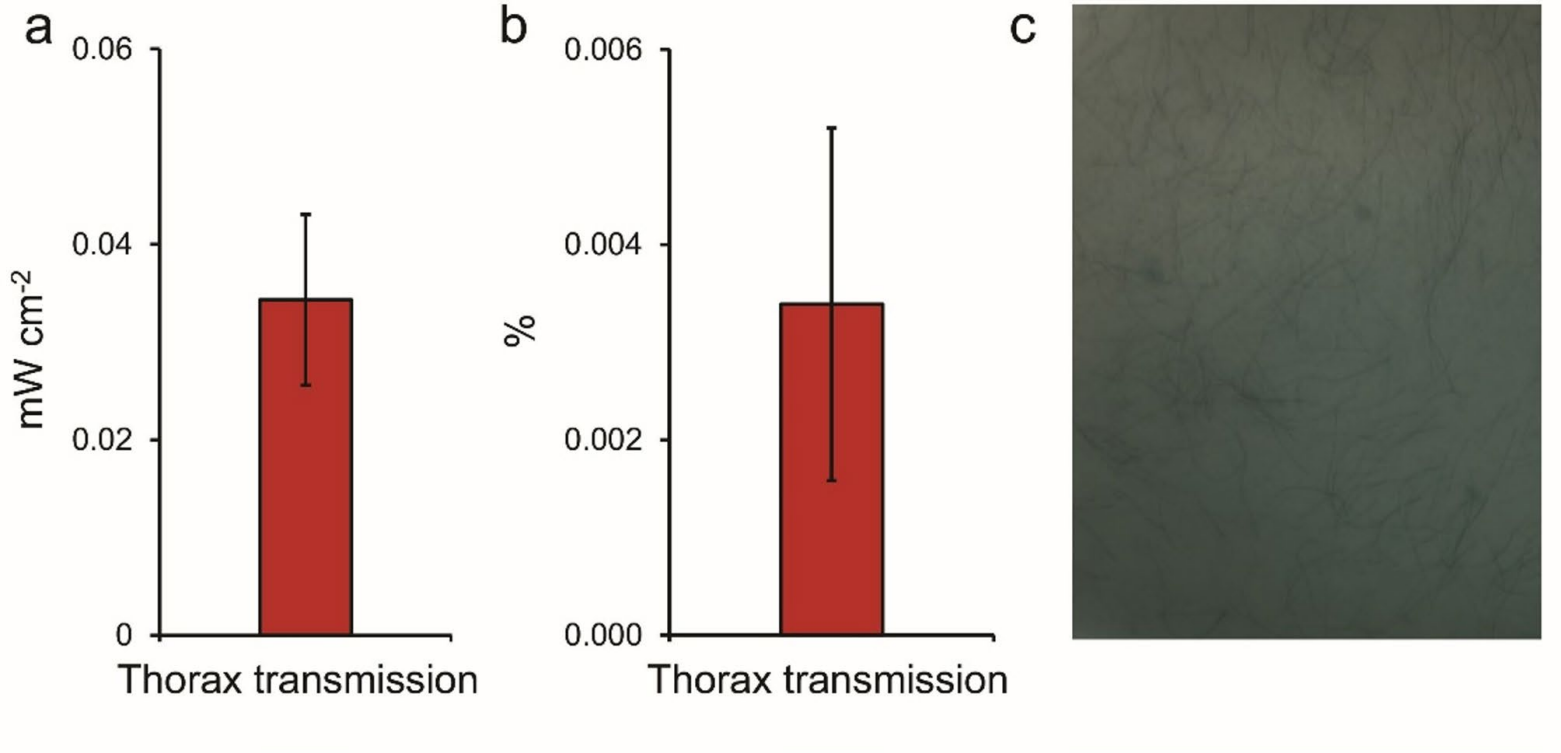
(a) Sunlight transmission through the thorax given as a percentage of sunlight (b), also in absolute values. (c). as imaged with a sensitive IR camera against the body. In all subjects sunlight could be measured following transmission through the thorax. Measurements were made between approximately 11am and 1pm in direct sunlight. The radiometer used to measure the energy was embedded in an aluminium box packed with aluminium foil to block stray light. A photograph from an infra-red sensitive camera showing the transmitted light is to the right. This was taken with a 10 s exposure, aperture f/8, speed ISO 100 and a focal length of 20 mm.

Subjects were exposed to direct sunlight around midday and measurements made of light transmission through the chest were made by placing a radiometer on their chest within an aluminium light tight box shield. In each of the subjects tested, exposure of the body surface to direct sunlight resulted in long wavelength light detection on the other side of the body. This is shown in Fig. 1. Figure 1a shows the absolute measurement of light through the thorax, Fig. 1b shows the percentage of light passing through and Fig. 1c, is an infra-red image of the light captured after its passage through the thorax. Hence, the body showed a degree of transparency to sunlight.

**Fig. 2:**
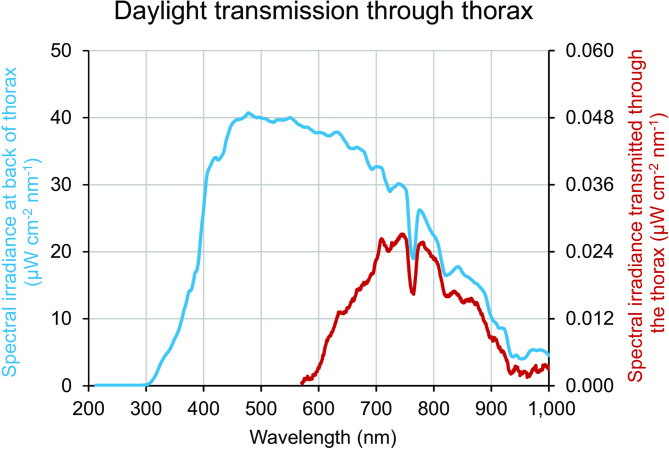
Body transmission of sunlight in a representative individual. Blue line is the spectrum of sunlight present during measurements of light through the thorax. Sunlight ranged from approximately 300 nm to > 1000 nm. The power of the light is given on the left Y axis. Red line is the spectrum of direct sunlight passing through the thorax was measured. This showed a mark long wavelength shift between approximately 600 nm to > 1000 nm. The energy of light passing through the thorax was very reduced and is given on the right-hand Y. The very low level of sunlight detected passing through the body is consistent with a large percentage of light being absorbed/scattered by the body.

An example of the spectrum of light transmitted through the thorax is shown in Fig. 2. The blue line is the spectrum of direct sunlight on the day in which transmission was measured. The measured sunlight spectrum ranges from approximately 300 nm to approximately 1000 nm. The transmitted light through the thorax is much reduced compared to direct sunlight and is longwave wavelength shifted spanning approximately 600 nm to approximately 1000 nm. Note the different scaling on the left and right Y axes that have been used so that both spectra can be presented together.

**Fig. 3:**
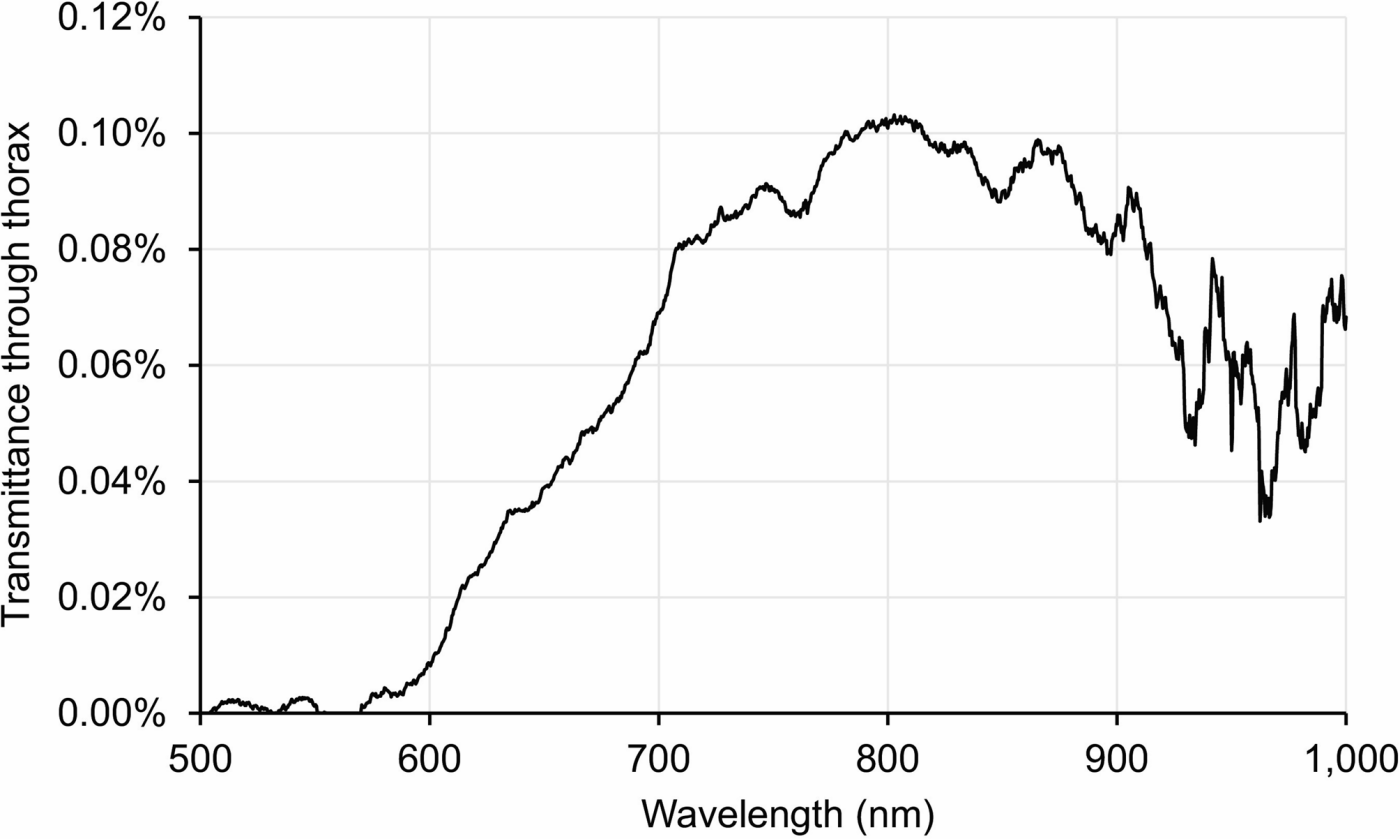
This curve is a ratio of the spectral irradiance through the thorax, relative to the spectral irradiance of daylight falling onto the thorax. This therefore provides an indication of which wavelengths are absorbed most strongly by the body, and which pass through. Note that the incident daylight is highly directional whereas the transmitted light is scattered in all directions; the small magnitude of the transmitted irradiance is due to it being spread over a larger area. There is very little transmission below 600 nm, with transmission increasing up to a peak in the range 800–875 nm. Instability above 930 nm is due to instrumentation.

In Fig. 3 the ratio of transmitted light to the sunlight spectrum is shown. This results in a peak in the region of 800 nm to approximately 870 nm. At 850 nm sunlight has a strength of approximately 17mW/cm^2^, while that measured after transmission through the chest had an average of approximately 5.6µW/cm^2^.

These results show that sunlight is transmitted through the body, but they likely suffer from a degree of noise as it is difficult to measure key metrics in environmental conditions lacking tight laboratory controls. Hence, two additional experiments were undertaken in laboratory conditions in darkness. The first used a thermal continuum source on the hand with the aim of identifying key absorbers around 850 nm in this thinner tissue that was approximately 3 cm thick. The second used a large LED panel source illuminating at a peak of 850 nm with half-power bandwidth of approximately 35 nm, which includes the range of the peak ratio shown in Fig. 2. This was undertaken to determine the impact of longer wavelengths found in sunlight on visual function.

**Fig. 4:**
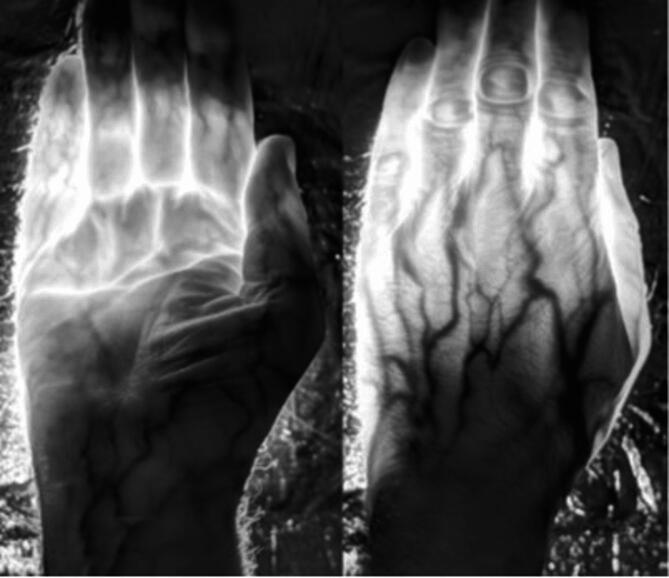
Light transmission through the hand from an 850 nm LED source. Because the tissues are relatively thin compared with the thorax it was possible to map the spectrum here against known biological absorbers. The images clearly show that deoxygenated blood is a key absorber. Also, bone can not be seen and hence is relatively transparent at these longer wavelengths.

**Fig. 5:**
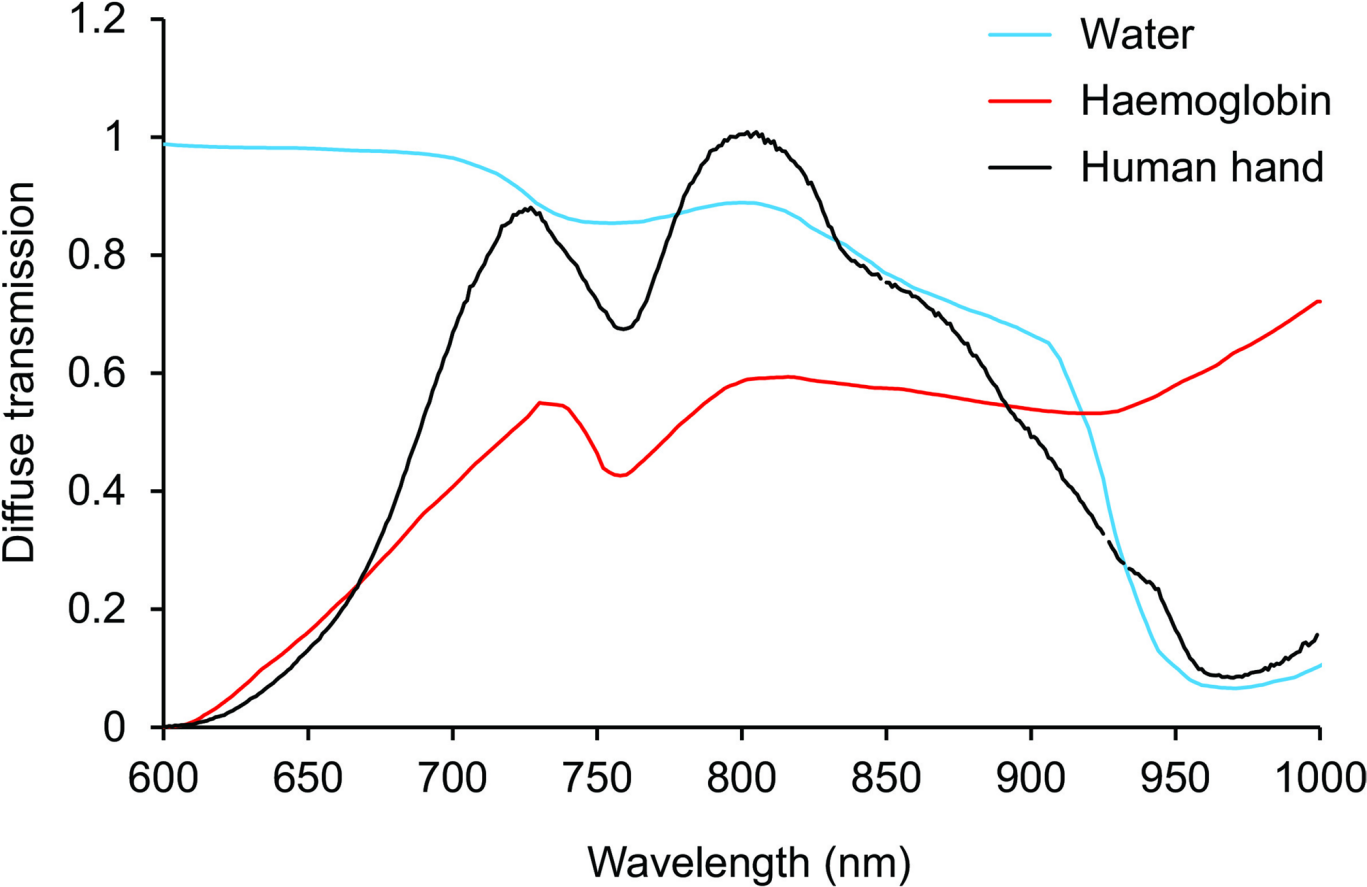
Spectral transmission of a daylight type source through the hand from a tungsten filament via an integrating sphere. The diffuse transmittance spectrum through the mid-palm of the human hand normalised to a peak of unity. The principal absorbers are haemoglobin at shorter, and water at longer wavelengths. This was measured with a Maya2000Pro spectrometer using an ISP-REF integrating sphere as a tungsten filament light source and a 500 μm diameter optical fibre (all from OceanInsight) to collect the transmitted light.

Figure 4 shows the transmission of light passing through the hand using an infrared sensitive camera. The tissue is very transmissive with only deoxygenated blood showing a degree of absorbance as the veins are clearly visible in black. Bones in the hand show no indication of absorbance. The spectrum of light passing through the hand is shown in Fig. 5.

The overall profile, shown in black, is similar to that seen in Fig. 2 for thorax transmission and covers a very similar spectral range. Outlined on the absorption profile are two principal absorbers, water in blue and haemoglobin in red. Water and haemoglobin both contribute to the dip seen around 750 nm. Water absorbance is a key contributor to the gradual decline seen from around 820 nm onwards. Other weak absorbers most likely contribute to the detailed spectral shape.

**Fig. 6:**
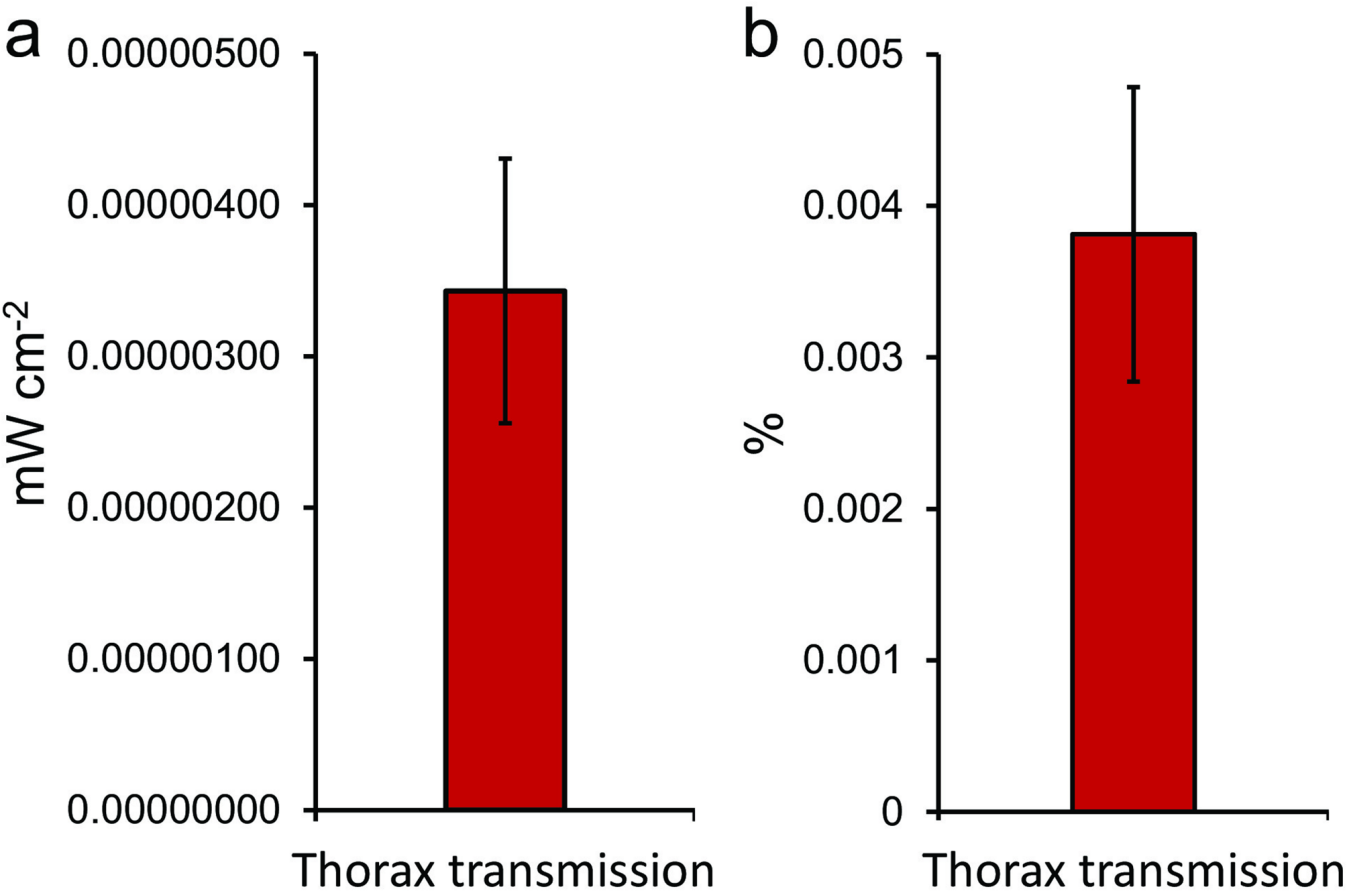
Light transmission through the chest from an 850 nm LED panel in laboratory controlled dark conditions. (a) shows the absolute light level and (b) the percentage. The proportion of this wavelength penetrating the body is similar to that in sunlight, but the absolute levels is much lower than sunlight. It was not possible to capture and infrared image or to plot the spectrum of transmitted light because the energy levels were so low.

Light in the 850 nm range that is found in sunlight shows a clear ability to penetrate deeply through the body and it is likely that such wavelengths improve mitochondrial function that likely impacts on physiological ability. We measured the light transmission of the thorax to the 850 nm panel in otherwise dark conditions. This is shown in Fig. 6. Figure 6a shows the absolute transmission while 6b shows the percentage transmission. An infrared image like that shown in Fig. 1c could not be obtained because there was not enough light. Hence, light from the 850 nm panel was transmitted through the body, as observed in sun light although at a lower energy level. Because the energy was low it was not possible to reliably plot its spectrum. However, we did explore the consequences of its transmission.

To assess this, we examined colour contrast detection thresholds in subjects exposed to the 850 nm panel. Previously we have used this sensitive metric to reveal improved human colour visual function following exposure to 670 nm light^7^. This psychophysical method was used in subjects exposed to the 850 nm in laboratory conditions to detect changes in visual thresholds along the both tritan (blue-yellow) and the protan (red-green) visual axis. 850 nm light was initially directed at subjects backs from 1.5 m with their eyes closed for 15 min. However, we noted that radiometric measurements of reflected light from walls and other surfaces from the light generated by the 850 nm panel were high and multidirectional. Hence, a subgroup had their heads wrapped in aluminium foil to block ocular exposure arising from reflections.

**Fig. 7:**
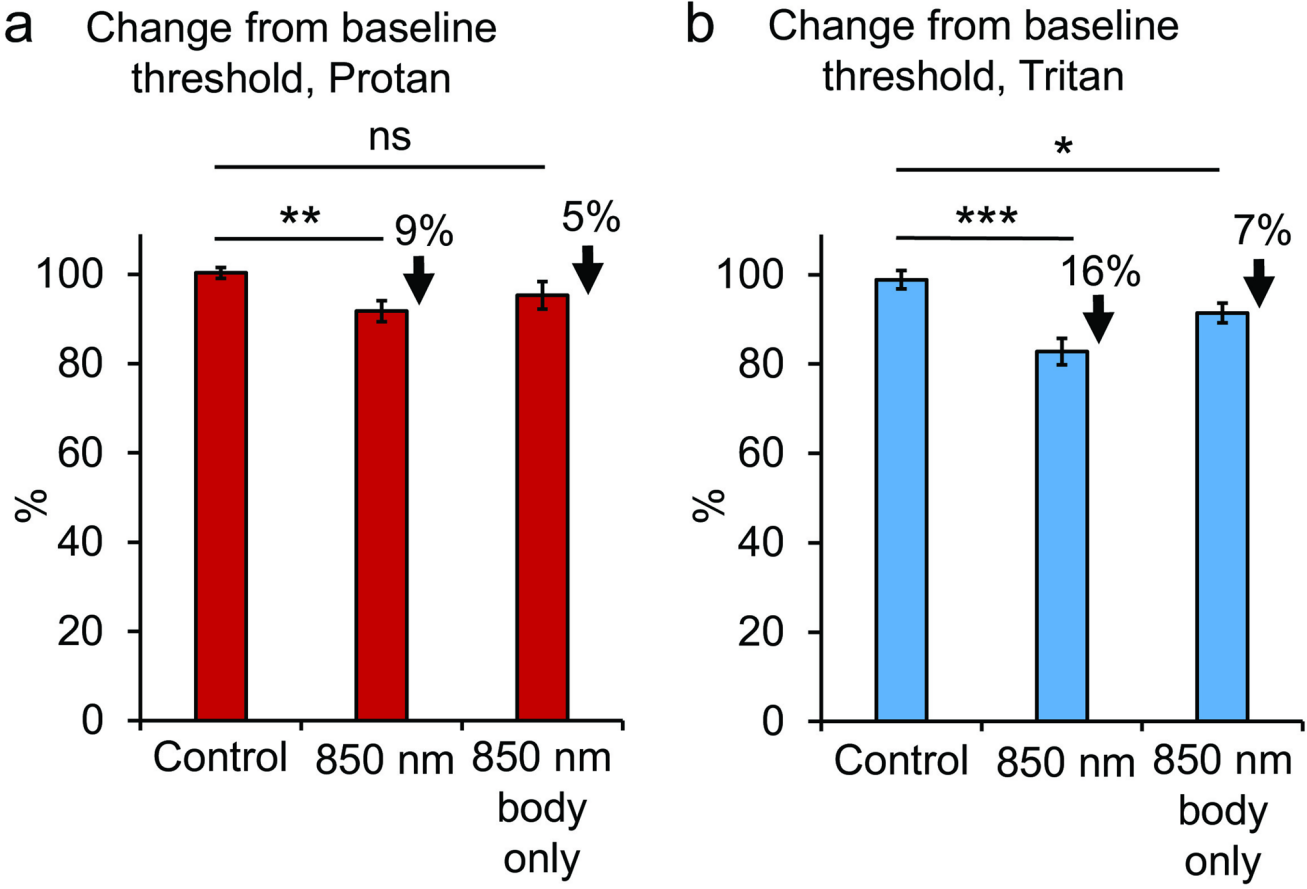
Colour contrast measurements made for protan (red) and tritan (blue) contrast sensitivities. (a)Thresholds for Protan contrast sensitivity were reduced by a significant 9% following 850 nm exposure to the back but although reduced when the head was shielded from the light this was not significant (body group only). (b) Thresholds for tritan are more long wavelength sensitive than protan. Thresholds here were reduced by a significant 16% with 850 nm exposure to the back and by a significant 7% when the head was shielded (body group only). These data confirm that light exposure to the body can improve visual function independent of ocular input. Abbreviations: ns, not significant. Statistical symbols * *p* < 0.05. ** *p* < 0.01. *** *p* < 0.001.

Figure 7 shows colour contrast threshold measurements made for protan and tritan. Thresholds for protan contrast sensitivity were reduced by a significant 9% (*p* = 0.0053) following 850 nm exposure to the back. Although they were also reduced when the head was shielded from the light, this was not significant (*p* = 0.1856). Thresholds for tritan are more sensitive than protan to long wavelength exposure, with relatively larger improvements following exposures as shown in previous studies using 670 nm^7^. Thresholds for tritan following 850 nm exposure were reduced by a significant 16% (*p* = 0.0003) with exposure to the back and by a significant 7% (*p* = 0.0351) when the head was shielded. These data confirm that light exposure to the body can improve visual function independent of ocular input, but to different degrees. With the head shielded the consequences of exposure must have been mediated by light transmitted through other regions of the body.

**Fig. 8:**
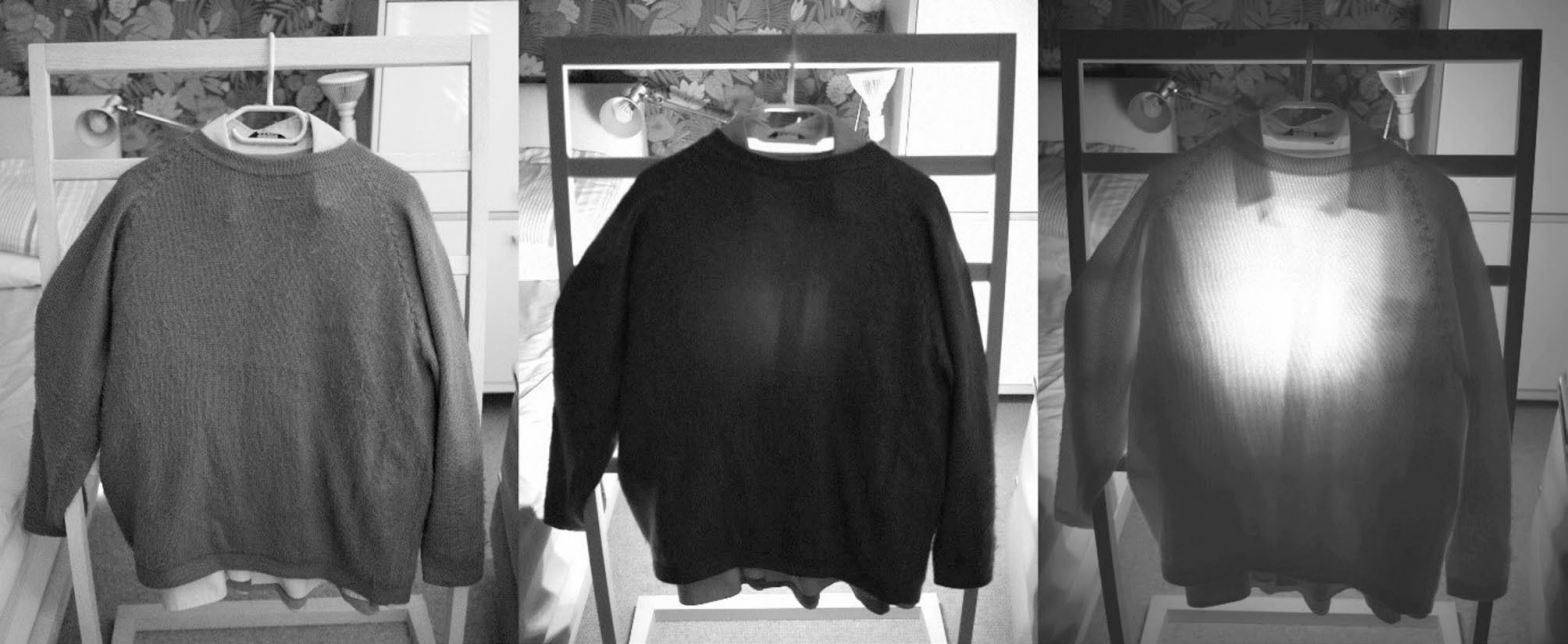
The transmission of light through clothing. Images were captured with a camera whose sensitivity covered the visual and infra-red range. Each panel contains an image of light transmission through a T-shirt, a shirt and a woolen jumper on a hanger. Left: image in ambient daylight; Middle: image backlit with an LED lamp both photographed in visible only light; Right: the same as the middle image but taken with an infrared (750–1000 nm) camera passing only the 850 nm light from a 54 W 660/850nm Candeer red light therapy LED lamp. This shows that the clothes (six layers in all) are nearly 100 times more transparent at 850 nm than in the visible. The answer is the NIR light penetrates normal indoor clothing quite effectively.

This study shows that longer wavelengths present in sunlight penetrate the body and can be measured after they pass through. Further, that these have the ability to improve visual function independent of ocular involvement. A natural question to arise is how this transparency changes with clothing and to what extent does clothing act as a barrier. As types of clothing vary widely depending on fabric type and weave, the question cannot realistically be answered quantitatively. However, Fig. 8 shows three images of the same common clothing items combined including a T-shirt, a shirt and a woollen jumper (6-layers in total) taken with a camera that has both normal and infra-red sensitivity. The first is taken in normal reflected light, the second with a light source in the human visual range behind the items and the third in infrared mode, with an 850 nm light source behind. The clothing shows a marked transparency to the 850 nm light source. Hence, clothing is unlikely to be a barrier to long wavelength penetration of the body and any subsequent functional improvements that may occur because of it.

## Discussion

This study shows that longer wavelengths in sunlight are transmitted through the body. When these are presented via NIR LEDs in a laboratory-controlled environment at much lower energies, they again have the ability to be transmitted through the body and also are associated with improved visual function independent of ocular exposure. Hence, body penetration by longer wavelengths impacts systemically. Longer wavelengths improve mitochondrial membrane potential and ATP production and improve function in a species conserved pattern that can impact on mobility, visual function and cognition, particularly in ageing^2–4,6,7^. In short lived animals they can extend average life span^15^.

Taken together, these data provide evidence of the importance of the full spectrum of sunlight for human health. They also highlight the potential dangers of the restricted spectra found in white LED lighting in the modern built environment that lacks longer wavelengths and whose output is generally restricted to around 400–650 nm. The absence of longer wavelengths from LED light sources may have implications for public health that should be addressed^16^.

This study is not the first to measure light penetrance through the human body, as Hart and Fitgerald^17^ made measurements in postmortem tissues. Our results generally agree with theirs, but none of their measurements were undertaken in sunlight and clearly none could be matched against psychophysical function.

The results on improved visual function with 850 nm are consistent with other studies that have used LED-based longer wavelengths to improve mitochondrial performance, particularly 670 nm, for which there are numerous studies^7^. But we note in data not reported here that 670 nm does not transmit across the human body, which is consistent with relatively shorter wavelengths having reduced biological penetrance. The increasing strength of water absorbance bands towards wavelengths longer than 900 nm (see Fig. 4) implies that only restricted spectral regions will allow penetrance above 1000 nm. In terms of overall outcome on visual function, there is little obvious difference between 670 nm and 850 nm, although they have not been tested under identical conditions. We do not know the relative merits of wavelengths between 670 nm and 900 nm, which remains to be explored.

It is known that improving mitochondrial function by illumination at a specific location results in distal as well as proximal effects. This is called the abscopal effect. It was noted scientifically by Durieux et al. who commented using C elegans “we find that mitochondrial perturbation in one tissue is perceived and acted upon by the mitochondrial stress response pathway in a distal tissue”^18^. Clinically it is recognised that targeted irradiation of a primary tumour can result in shrinkage of secondary tumours in distal tissues^19^. Such patterns are present in many reports using longer wavelengths to improve mitochondrial function, and they show that it triggers a widely communicated response^13,20,21^. A key example of this is the exposure of 670 nm light to a limited skin region that results in a significant reduction in challenged blood sugars^11^. The authors argue that this arises from increased carbohydrate demand from stimulated mitochondria. But the size of the effect can only arise if mitochondria in a much wider region than illuminated are upregulated and increase demand for blood sugars.

The route of the abscopal effect is unclear. However, 670 nm irradiation in mice results in changes in patterns of serum cytokine expression that may be part of the pathway for systemic effects^14^ If correct, then depth of light penetration arising from longer wavelengths may not be a significant variable because all that is needed is a triggering effect on targeted tissues including the skin, although the energy, wavelength and time of day regulating this are unknown. Improved human vision with 670 nm light can be obtained with only 3 min exposure within 3 h of application^7^. In insect experiments where greater numbers can be used, there is a clear tiggering effect in exposure times at around 1 min, with no evidence for a dose response function^22,23^. Given the highly conserved nature of long wavelength exposure on mitochondrial performance, one might expect the same in human experiments. Understanding the limits of these variables are of importance in understanding needs for human health.

Of equal importance is the identification of the absorbers of these wavelengths and their mode of action in improving physiological function. There have been no systematic attempts to define an action spectrum for the effect of the NIR light at different wavelengths. Hence, in spite of its relatively shallow body penetration, use of 670 nm remains common although it is limited because it can dominate the visual environment. We have noted some of the absorbers at 850 nm but there is still no clear understanding of the complexities of infra-red absorption. Hence, we should consider the possibility of many weak absorbers at different wavelengths combining to influence the interactions between biomolecules, especially those comprising the mitochondrial Electron Transport Chain (ETC).

Individual NIR photons at wavelengths near the maximum of body transmission carry about forty times the kinetic energy of a biomolecule at body temperature (~ 310 K). On absorption, these photons can accelerate redox reaction rates in the ETC and so increase ATP production. By adding the extra energy as a radiative excitation to a low-lying electronic or vibrational state rather than heat, this process operates without an excessive increase in tissue temperature. Such NIR-accelerated processes are well-known as ‘Green Chemistry’ in chemical production^24^ but we propose that they may be important for mitochondrial function in living tissues.

Such a picture would be seen as a real-time effect limited to the period when the NIR photons were available. There is evidence however that short periods of illumination have a lasting effect on mitochondrial function extending for multiple days following the dose^7,15^. This suggests that the NIR is generating a long-lasting photocatalyst. This has not been identified but could be associated with the orchestration of the mechanisms that control the balance of ROS and antioxidants that maintain full mitochondrial function^25^.

In spite of the potential parallels that can be drawn between sunlight and selective exposure to long wavelength ranges, there are numerous key differences. Both may have the ability to influence blood glucose levels, but sunlight has a continuous spectrum and is present for extended periods, while experimental long wavelength delivery has a reduced spectral range and is often delivered at relatively high energy and for short periods. Hence, it is possible that 670 nm and 850 nm exposure release a transitory mechanism that is permanently expressed in sunlight but at a lower more constant level.

### The shift from sunlight to leds in the built environment

There has been a major change in the human experience of light since the introduction of LED lighting in the early twenty first century. While evolution has taken place under sunlight, the human experience is now very different. The move to a built environment has taken humans away from their evolutionary history in terms of reducing sunlight exposure, but this was initially mitigated by two issues. Both fire light and light from older incandescent lighting have significant longer wavelength components and hence similarities to solar light. This is not the case for standard LED lighting used in buildings that has come to dominate over the last 20 years. In white LED lighting whether warm or cool there are few if any wavelengths over 650 nm and the dominant wavelength is blue at approximately 450 nm: 420–450 nm is known to undermine mitochondrial function, particularly in the absence of IR^9,26,27^. Such wavelengths are intense in lighting units with colour temperature of 4000–5000 K. These wavelengths not only undermine mitochondrial performance, but also have an immediate effect significantly increasing human heart rate and significantly reducing blood pressure^28^. This is most likely mediated by surface skin absorbance. Hence, as with NIR used to control blood sugars^11^skin absorbance may be key to some critical physiological processes that do not need deep light penetrance. These factors are largely overlooked but may raise issues of concern for public health.

## Data Availability

The data sets used are and/or analysed during the current study are available from the corresponding author on reasonable request.
